# Supplementary material for: Sleep in People Experiencing Homelessness Under Different Conditions and Seasons
Source: bioRxiv. 2025 Jan 26:2025.01.23.634551. Preprint. [Version 1] doi: 10.1101/2025.01.23.634551 (PMC11785220; doi:10.1101/2025.01.23.634551)
Supplement: Supplement 1 [file NIHPP2025.01.23.634551v1-supplement-1.pdf]

bioRxiv preprint doi: https://doi.org/10.1101/2025.01.23.634551; this version posted January 26, 2025. The copyright holder for this preprint (which was not certified by peer review) is the author/funder, who has granted bioRxiv a license to display the preprint in perpetuity. It is made available under aCC-BY 4.0 International license.

Supplementary information for:

Sleep in People Experiencing Homelessness Under Different Conditions and Seasons

Alicia Rice[1], Leandro P. Casiraghi[1,a], Cristina Gildee[2], Zack W. Almquist[3], Amy Hagopian[4], Melanie A. Martin[4], Horacio O. de la Iglesia[1]

This file contains Tables S1-S7 and Fig. S1.

Table S1.a. Sleep parameters linear model estimates for summer

**Summer**						
	**Community (vs. Housed)**	**Mean ± SEM**	**Estimate (hours)**	**Std. Error**	**t-Value**	**p-Value**
**Sleep Onset**	Overnight Shelter	23:33 ± 00:14	-0.5800	1.2562	-0.462	0.648
	Continuous-Occupancy Shelter	01:04 ± 00:44	0.7808	1.4260	0.548	0.589
	Tent	23:09 ± 00:47	-0.7310	1.2253	-0.597	0.556
	Tiny House	00:26 ± 00:47	0.4748	1.2043	0.394	0.697
	Housed	23:35 ± 00:52				
**Sleep Offset**	Overnight Shelter	06:56 ± 00:15	-1.832	1.0813	-1.694	0.1026
	Continuous-Occupancy Shelter	08:18 ± 00:55	-0.7174	1.2275	-0.584	0.5641
	Tent	07:27 ± 00:35	-0.7608	1.0547	-0.721	0.4774
	Tiny House	07:28 ± 00:42	-0.9761	1.0366	-0.942	0.3554
	Housed	07:21 ± 00:29				
**Midsleep**	Overnight Shelter	03:15 ± 00:11	-1.206	1.1171	-1.080	0.291
	Continuous-Occupancy Shelter	04:41 ± 00:49	0.0317	1.2681	0.025	0.980
	Tent	03:18 ± 00:40	-0.7459	1.0896	-0.685	0.500
	Tiny House	03:57 ± 00:42	-0.2507	1.0709	-0.234	0.817
	Housed	03:27 ± 00:40				
**Sleep Duration**	Overnight Shelter	7h 23min ± 18min	-1.2519	0.7093	-1.765	0.0898
	Continuous-Occupancy Shelter	7h 14min ± 16min	-1.4982	0.8052	-1.861	0.0746
	Tent	8h 18min ± 19min	-0.0297	0.6918	-0.043	0.9661
	Tiny House	7h 1min ± 26min	-1.4510	0.6799	-2.134	**0.0428**
	Housed	7h 46min ± 24min				

Table S1.b. Sleep parameters linear model estimates for winter

**Winter**						
	**Community (vs. Housed)**	**Mean ± SEM**	**Estimate (hours)**	**Std. Error**	**t-Value**	**p-Value**
**Sleep Onset**	Overnight Shelter	23:28 ± 00:17	0.2194	0.6396	0.343	0.7335
	Continuous-Occupancy Shelter	23:50 ± 00:25	0.3264	0.8499	0.384	0.7032
	Tent	23:11 ± 1:06	-0.1691	0.7639	-0.221	0.8260
	Tiny House	23:54 ± 00:33	0.7926	0.5815	1.363	0.1814
	Housed	23:07 ± 00:19				
**Sleep Offset**	Overnight Shelter	06:28 ± 00:20	-1.2654	0.6851	-1.847	0.073
	Continuous-Occupancy Shelter	06:41 ± 00:27	-0.8939	0.9104	-0.982	0.333
	Tent	06:59 ± 1:12	-0.6638	0.8183	-0.811	0.423
	Tiny House	07:03 ± 00:36	-0.5572	0.6230	-0.894	0.377
	Housed	07:31 ± 00:12				
**Midsleep**	Overnight Shelter	02:58 ± 00:17	-0.5230	0.6369	-0.821	0.4170
	Continuous-Occupancy Shelter	03:15 ± 00:24	-0.2837	0.8464	-0.335	0.7394
	Tent	03:06 ± 1:09	-0.4164	0.7607	-0.547	0.5875
	Tiny House	03:29 ± 00:34	0.1177	0.5791	0.203	0.8401
	Housed	03:19 ± 00:14				
**Sleep Duration**	Overnight Shelter	6h 59min ± 15min	-1.4848	0.3663	-4.053	**0.0003**
	Continuous-Occupancy Shelter	6h 50min ± 23min	-1.2202	0.4868	-2.507	**0.0168**
	Tent	7h 48min ± 7min	-0.4948	0.4375	-1.131	0.2656
	Tiny House	7h 9min ± 15min	-1.3498	0.3331	-4.052	**0.0003**
	Housed	8h 24min ± 16min				

bioRxiv preprint doi: https://doi.org/10.1101/2025.01.23.634551; this version posted January 26, 2025. The copyright holder for this preprint (which was not certified by peer review) is the author/funder, who has granted bioRxiv a license to display the preprint in perpetuity. It is made available under aCC-BY 4.0 International license.

Table S2. Waveform morning and evening cumulative counts one-way ANOVA

**Summer**				
	**Fixed Effect**	**Df**	**F-value**	**p-value**
Morning (7:00-10:00)	Community	4	2.9	**0.0406**
Evening (19:00-21:30)	Community	4	2.584	0.0596

**Winter**				
	**Fixed Effect**	**Df**	**F-value**	**p-value**
Morning (7:00-9:00)	Community	4	4.365	**0.0051**
Evening (20:00-22:00)	Community	4	2.068	0.1030

Table S3.a. SRI one-way ANOVA

	**Fixed Effect**	**Df**	**F-value**	**p-value**
**Summer**	Community	4	2.804	**0.0456**
**Winter**	Community	4	8.579	**< 0.0001**

Table S3.b. Dunnett's Test for SRI

	**Community (vs. Housed)**	**p-value**
**Summer**	Overnight Shelter	0.9896
	Continuous-Occupancy Shelter	0.5819
	Tent	0.2461
	Tiny House	0.2139
**Winter**	Overnight Shelter	0.9705
	Continuous-Occupancy Shelter	**0.0327**
	Tent	**0.0003**
	Tiny House	**0.0005**

Table S4.a. Activity During Sleep one-way ANOVA

	**Fixed Effect**	**Df**	**F-value**	**p-value**
**Summer**	Community	4	10.7	**< 0.0001**
**Winter**	Community	4	6.791	**0.0003**

Table S4.b. Dunnett's Test for Activity During Sleep

	**Community (vs. Housed)**	**p-value**
**Summer**	Overnight Shelter	0.8665
	Continuous-Occupancy Shelter	0.6534
	Tent	0.1342
	Tiny House	**0.0011**
**Winter**	Overnight Shelter	0.2550
	Continuous-Occupancy Shelter	0.7850
	Tent	**< 0.0001**
	Tiny House	**0.0063**

bioRxiv preprint doi: https://doi.org/10.1101/2025.01.23.634551; this version posted January 26, 2025. The copyright holder for this preprint (which was not certified by peer review) is the author/funder, who has granted bioRxiv a license to display the preprint in perpetuity. It is made available under aCC-BY 4.0 International license.

Table S5.a. Intraindividual variance one-way ANOVA

**Summer**				
	**Fixed Effect**	**Df**	**F-value**	**p-value**
Sleep Onset	Community	4	1.09	0.3813
Sleep Offset	Community	4	0.882	0.4877
Midsleep	Community	4	1.599	0.5395
Sleep Duration	Community	4	1.379	0.2033

**Winter**				
	**Fixed Effect**	**Df**	**F-value**	**p-value**
Sleep Onset	Community	4	4.544	**0.0040**
Sleep Offset	Community	4	2.09	0.1001
Midsleep	Community	4	3.109	**0.0256**
Sleep Duration	Community	4	1.0514	**0.0049**

Table S5.b. Dunnett's test for intraindividual variance analysis

**Winter**		
	**Community (vs. Housed)**	**p-value**
**Sleep Onset**	Overnight Shelter	0.9892
	Continuous-Occupancy Shelter	0.9965
	Tent	**0.0051**
	Tiny House	0.0988
**Sleep Offset**	Overnight Shelter	1.0000
	Continuous-Occupancy Shelter	0.7848
	Tent	0.1168
	Tiny House	0.1583
**Midsleep**	Overnight Shelter	0.9898
	Continuous-Occupancy Shelter	0.9982
	Tent	**0.0332**
	Tiny House	0.1764
**Sleep Duration**	Overnight Shelter	0.7306
	Continuous-Occupancy Shelter	**0.0495**
	Tent	**0.0113**
	Tiny House	**0.0109**

bioRxiv preprint doi: https://doi.org/10.1101/2025.01.23.634551; this version posted January 26, 2025. The copyright holder for this preprint (which was not certified by peer review) is the author/funder, who has granted bioRxiv a license to display the preprint in perpetuity. It is made available under aCC-BY 4.0 International license.

Table S6. 50-lux exposure times linear model estimates

	**Fixed Effect**	**Df**	**F-value**	**p-value**
First Exposure	Season	1	**8.7341**	**0.0044**
	Community	4	6.9813	**0.0001**
	Gender	3	4.6709	**0.0052**
	Season:Community	4	2.2880	0.0697
Last Exposure	Season	1	17.1610	**0.0001**
	Community	4	15.9751	**< 0.0001**
	Gender	3	1.6547	0.1858
	Season:Community	4	1.6711	0.1678
Duration	Season	1	21.2113	**< 0.0001**
	Community	4	17.3567	**< 0.0001**
	Gender	4	3.5704	**0.0188**
	Season:Community	4	2.3437	0.0643

Table S7. Participant characteristics

	Tiny House		Tent City		Overnight Shelter		Continuous-Occupancy Shelter		Housed	
	Winter n = 14	Summer n = 10	Winter n = 9	Summer n = 12	Winter n = 9	Summer n = 10	Winter n = 7	Summer n = 7	Winter n = 14	Summer n = 6
Recording dates	1/23/2023-2/22/2023	9/5/2022-10/17/2022	1/23/2023-2/22/2023	9/5/2022-10/17/2022	2/7/2019-3/6/2019	9/5/2018-10/4/2018	1/23/2023-2/22/2023	9/5/2022-10/17/2022	2/15/2021-4/7/2021	9/26/2023-10/14/2023
**Self-Reported Gender (%)**										
Female	36	30	33	33	22	10	0	14	43	67
Male	50	70	67	58	67	80	86	71	50	33
Non-binary or transgender	14	0	0	0	11	10	14	14	0	0
Other or missing	0	0	11	8	0	0	0	0	7	0
**Self-Reported Race (%)***										
Caucasian	57	60	78	67			86	86	36	67
Black	35	20	0	56			14	14	14	0
Asian	0	10	0	0			0	0	7	17
Hispanic	0	0	0	0			0	0	35	17
American Indian	14	20	11	14			14	0	0	0
Other or missing	0	0	11	0			14	0	7	0
**Age (years): mean [range]**	43 [28-60]	48 [31-63]	44 [35-55]	44 [24-65]	22 [19-26]	23 [18-26]	43 [32-58]	44 [26-58]	38 [24-68]	30 [22-58]
**Excluded participants (n)**	2	2	4	7	1	0	2	2	0	2

*Participants may have reported more than one race, so that the total percent could be greater than 100%

** Overnight shelter race was not self-identified

bioRxiv preprint doi: https://doi.org/10.1101/2025.01.23.634551; this version posted January 26, 2025. The copyright holder for this preprint (which was not certified by peer review) is the author/funder, who has granted bioRxiv a license to display the preprint in perpetuity. It is made available under aCC-BY 4.0 International license.

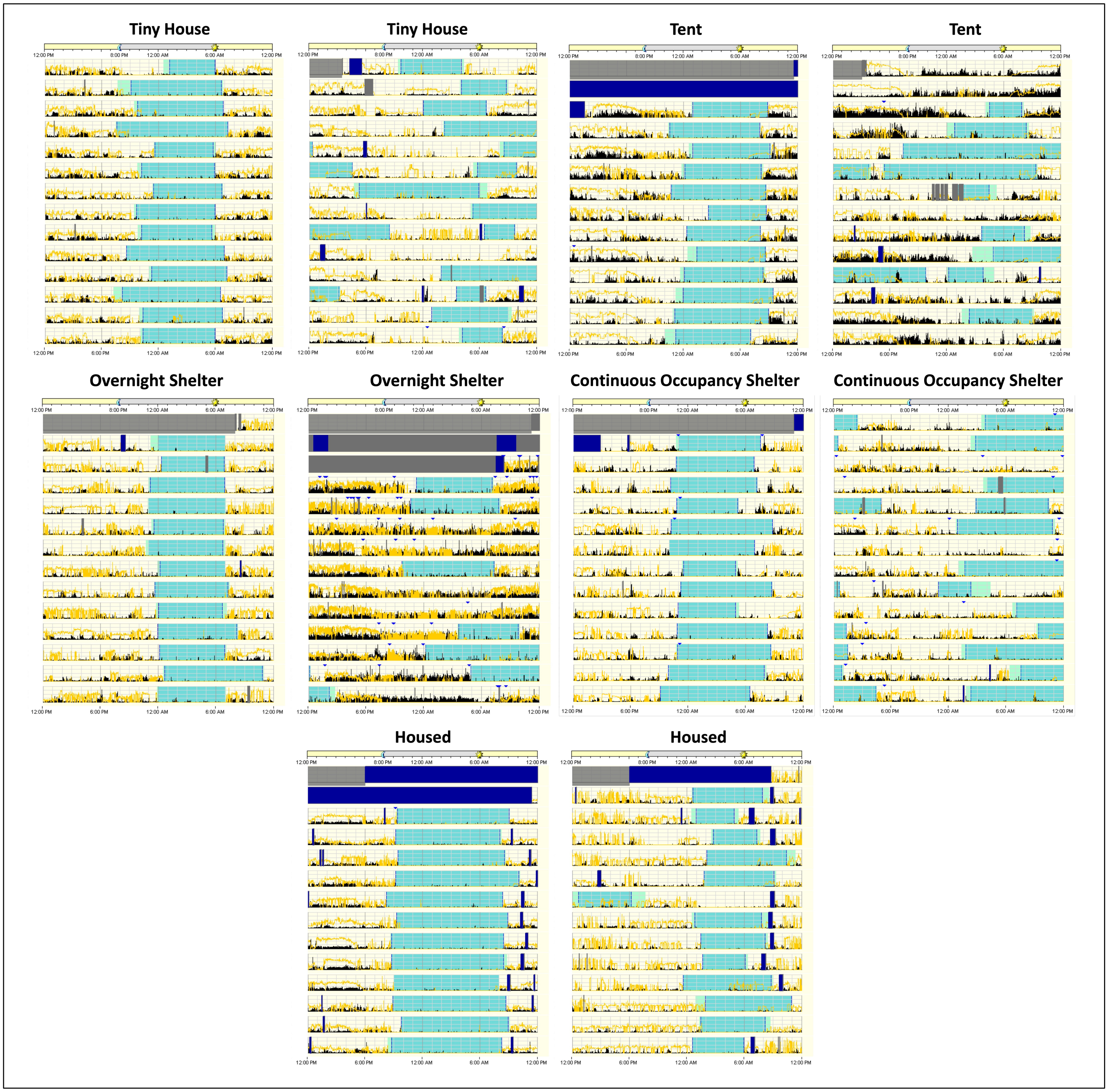

Figure S1. Actiwatch generated 24-hour actograms (12:00–12:00) of two participants from each community. The two actograms are representative examples from opposite ends of the spectrum between regular and most irregular sleep for each community. Sleep bouts are shown in light blue shaded sections, activity level is represented by black lines, and white light exposure by yellow lines.
